# Targeted delivery of harmine to xenografted human pancreatic islets promotes robust cell proliferation

**DOI:** 10.1038/s41598-022-19453-5

**Published:** 2022-11-09

**Authors:** Swati Mishra, Philip R. Streeter

**Affiliations:** 1 Brenden-Colson Center for Pancreatic Care, Oregon Health and Science University, Portland, OR USA; 2 Department of Pediatrics, Papé Family Pediatric Research Institute, Oregon Stem Cell Center, Oregon Health and Science University, Portland, OR USA

**Keywords:** Biomarkers, Diseases, Medical research, Chemistry, Materials science, Nanoscience and technology, Imaging, Microscopy

## Abstract

Type 1 diabetes (T1D) occurs as a consequence of the autoimmune destruction of insulin-producing pancreatic beta (β) cells and commonly presents with insulin deficiency and unregulated glycemic control. Despite improvements in the medical management of T1D, life-threatening complications are still common. Beta-cell replication to replace lost cells may be achieved by using small-molecule mitogenic drugs, like harmine. However, the safe and effective delivery of such drugs to beta cells remains a challenge. This work aims to deploy an antibody conjugated nanocarrier platform to achieve cell-specific delivery of candidate therapeutic and imaging agents to pancreatic endocrine cells. We approached this goal by generating core–shell type micellar nanocarriers composed of the tri-block copolymer, Pluronic®F127 (PEO_100_–PPO_65_–PEO_100_). We decorated these nanocarriers with a pancreatic endocrine cell-selective monoclonal antibody (HPi1), with preference for beta cells, to achieve active targeting. The PPO-based hydrophobic core allows encapsulation of various hydrophobic cargoes, whereas the PEO-based hydrophilic shell curbs the protein adhesion, hence prolonging the nanocarriers' systemic circulation time. The nancarriers were loaded with quantum dots (QDots) that allowed nanocarrier detection both in-vitro and in-vivo. In-vitro studies revealed that HPi1 conjugated nanocarriers could target endocrine cells in dispersed islet cell preparations with a high degree of specificity, with beta cells exhibiting a fluorescent quantum dot signal that was approximately five orders of magnitude greater than the signal associated with alpha cells. In vivo endocrine cell targeting studies demonstrated that the HPi1 conjugated nanocarriers could significantly accumulate at the islet xenograft site. For drug delivery studies, the nanocarriers were loaded with harmine. We demonstrated that HPi1 conjugated nanocarriers successfully targeted and delivered harmine to human endocrine cells in a human islet xenograft model. In this model, targeted harmine delivery yielded an ~ 41-fold increase in the number of BrdU positive cells in the human islet xenograft than that observed in untreated control mice. By contrast, non-targeted harmine yielded an ~ 9-fold increase in BrdU positive cells. We conclude that the nanocarrier platform enabled cell-selective targeting of xenografted human pancreatic endocrine cells and the selective delivery of the hydrophobic drug harmine to those cells. Further, the dramatic increase in proliferation with targeted harmine, a likely consequence of achieving higher local drug concentrations, supports the concept that targeted drug delivery may promote more potent biological responses when using harmine and/or other drugs than non-targeting approaches. These results suggest that this targeted drug delivery platform may apply in drug screening, beta cell regenerative therapies, and/or diagnostic imaging in patients with type 1 diabetes.

## Introduction

Type 1 diabetes (T1D) is an autoimmune disorder characterized by T-cell mediated insulin-producing beta cell loss leading to insulin deficiency and unregulated blood glucose levels^1^. Currently, 1.9 million individuals are living with T1D in the United States, and it affects approximately 9 million people worldwide^2,3^. While recent advancements in insulin therapy and the latest blood glucose monitoring technologies allow patients to measure their blood glucose levels more accurately so they may achieve optimal glycemic control, T1D associated chronic complications like ketoacidosis, heart attack, stroke, nephropathy, retinopathy, neuropathy, and hypoglycemia are still common^4^. In addition, more than 40% of patients receiving insulin therapy eventually become unaware of hypoglycemia^5^. Since experiencing frequent and prolonged hypoglycemic episodes can be potentially fatal, allogeneic transplantation of healthy pancreatic Islets of Langerhans is used as a last resort treatment to temporarily restore endogenous insulin production in such patients^6–8^. Nonetheless, limited donor availability to produce enough islet cells to achieve normoglycemia and long-term dependence on anti-rejection immunosuppressive drugs limit the large-scale implementation of this approach^9,10^. Therefore, it is crucial to develop an alternative therapeutic intervention to restore insulin production in patients with T1D. In this context, the identification of small-molecule drugs capable of enhancing the proliferation of human pancreatic beta cells in vivo (e.g., serpin b, harmine, and its derivatives) represents a breakthrough in potential therapeutic approaches^11–13^. However, currently, there is no effective method available to selectively deliver these highly potent mitogenic drugs to pancreatic endocrine cells or their subsets.

The overarching goal here is to regenerate functional beta cell mass from residual beta cells in type 1 diabetic pancreas. Thus, the study focuses on the cell-selective delivery of proliferation-inducing small molecules to pancreatic endocrine cells using antibody conjugated "actively targeted" nanocarriers. Focusing on human pancreatic islet and islet cell subset detection, Dorrell and colleagues developed monoclonal antibodies (mAbs) that react with cell surface molecules on discrete cell subsets in the human pancreas^14^. With the help of immunohistochemical staining, it has been demonstrated that these reagents efficiently target xenografted human endocrine cells in vivo (PRS, unpublished).

In recent years, mAbs have shown tremendous clinical success in cancer cell targeting and delivery of chemotherapeutic drugs specifically to solid tumors, which has led to the development of better diagnostic and therapeutic tools in the field of cancer research^15–18^. Similarly, diabetes researchers have been striving to precisely deliver therapeutic cargos to pancreatic islets and islet cell subsets to manipulate their properties and function. However, monoclonal antibodies have not yet been successfully used to target pancreatic islets for non-invasive imaging or therapeutic applications, mainly due to the unavailability of cell specific antibodies. Moreover, due to the limited volume of islet cells (< 2% of the total pancreatic volume) and low target expression levels, the targeting moiety must be specific for the pancreatic endocrine cells to target pancreatic islets successfully^19,20^. Within this frame of reference, Moore et al. could demonstrate excellent beta cell-specific accumulation of IC2 antibody conjugated radioactive and exendin 4 conjugated iron oxide based imaging probes in ex vivo imaging of the excised murine pancreata. Monoclonal antibody IC2 and exendin 4 respectively reacted with glucagon-like peptide 1 (GLP-1) receptors and an unknown molecule present on beta cells^20^. Subsequently, Balhuizen et al. have developed high-affinity camelid single-domain antibody (nanobody) targeting human Dipeptidyl-Peptidase 6 (DPP6) that localizes only in beta and alpha cells within the pancreas. The radiolabelled nanobodies demonstrated successful non-invasive visualization of DPP6-expressing cells transplanted in immunodeficient mice by SPECT/CT^21^.

**Figure 1:**
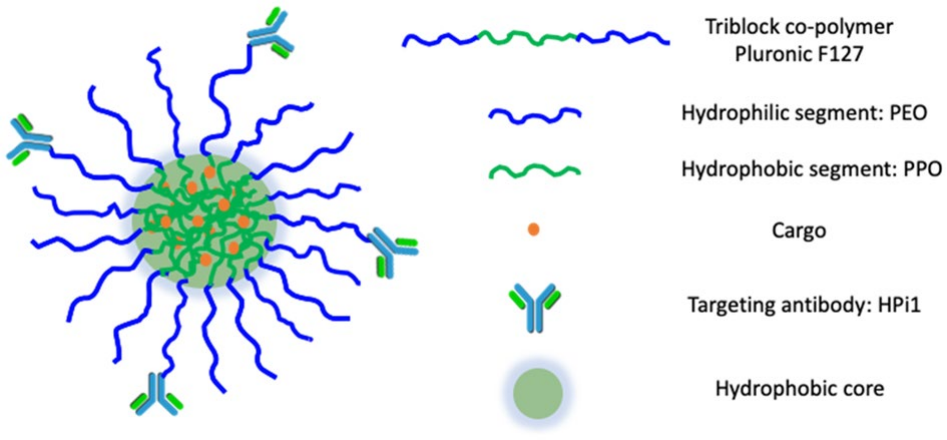
Schematic structure of cell type selective cargo encapsulating nanocarriers in aqueous solution. *PEO* polyethylene oxide, *PPO* polypropylene oxide.

In our studies, to achieve cell-type selective targeting, nanocarriers were conjugated to a pancreatic endocrine cell-selective monoclonal antibody (HPi1). HPi1 preferntially reacts to beta cells and a lesser extent with alpha cells. As shown in the schematic (Fig. 1), the nanocarrier platform described here consists of 1) a cell-selective targeting component, the monoclonal antibody HPi1, to facilitate active targeting of pancreatic beta cells, 2) a Pluronic F127 based nanocarriers that can accommodate a variety of hydrophobic cargo, such as imaging (quantum dots) or therapeutic agents (Harmine) and 3) cargo, allowing assessment of effective targeting, cargo delivery, and promotion of biological responses.

Overall, this nanocarrier platform has allowed us to explore antibody-mediated targeting and delivery of cell proliferating small molecule drug harmine through active targeting to human pancreatic endocrine cells transplanted in mice. This combination of features is anticipated to enable higher drug delivery efficiency, improve therapeutic efficacy, and minimize off-target side effects or toxicities. This nanocarrier platform may have application in the targeted delivery of various therapeutic and/or regulatory agents; such agents could be used to prevent beta cell apoptosis; promote beta cell regenerative processes; reduce the immunogenicity of beta cells or alter cellular phenotype and function.

## Materials and methods

### Materials

Pluronic F127 (P305 500 GM) was purchased from Anatrace Products LLC (Maumee, OH, USA). The core–shell type CdSe/ZnS (CZ520-25) green fluorescence and CuInS/ZnS (CIS750-25) near-infrared quantum dots were purchased from NNCrystal US Corporation (Fayetteville, AR, USA). The quantum dot formulations were obtained in chroform from the manufacturer. Harmine and Harmine hydrochloride salts were purchased from Sigma Aldrich (St. Louis, MO, USA). Benzene anhydrous (Fisher Chemicals Cat#B4121), nitrophenyl chloroformate (Cat#AC170800250), diethyl ether (Cat#AC176830010), and HPLC grade water (Cat#W5N-119) were used as received from Thermo Fisher Scientific, Inc. (Waltham, MA, USA). Phosphate buffer saline solution without calcium magnesium (Cat#SH30256.02), fetal bovine serum (FBS; Cat#SH30396.02HI), and trypsin (Cat#SH30236.01) were purchased from HyClone Laboratories Inc. (Logan, UT, USA). Corning® cellgro® CMRL media (Cat#15-110-CV) was used for islet cell preparations.

### Human pancreatic islet cell preparation

The Integrated Islet Distribution Program (IIDP, City of Hope) (https://iidp.coh.org) provided human islet samples for in vitro (less than 80% pure) and in vivo (more than 95% pure) studies. For the preparation of single-cell suspensions, islets were washed with 1 × PBS before incubation in 3 mL of 0.05% trypsin–EDTA per 5000 islet equivalent (IEQ) in a 15 ml falcon tube. Islets were digested for 10–12 min at 37 °C. Every 3 min the islets were gently dispersed using p1000 micro pipette. Subsequently, CMRL media supplemented with 10% FBS was slowly added to the enzyme dispersed cells to inhibit trypsin activity. Cells were then washed and resuspended in CMRL media supplemented with 10% FBS before incubation with nanocarriers.

### Antibodies

The human pancreatic endocrine cell reactive mouse mAb, HPi1 (Clone designation: HIC0-4F9), and isotype-matched negative control mouse mAb HPDAC1-6D2 (6D2; no reactivity with normal human pancreatic cells or with mouse cells) were used in this investigation.

For conjugation to nanocarriers, these mAbs were isolated from culture supernatants using Pierce™ protein G immobilized beaded agarose resin (Thermo Fisher Scientific, Waltham, MA) as per the manufacturer's guidelines. Purified mAb was buffer exchanged into PBS, filter-sterilized, and stored at 4 °C until used. For the xenograft immunolabeling, tissue sections were stained with 200 μl primary antibody HPi2 (Clone designation: HIC1-2B4) supernatant, which was detected with FITC conjugated goat anti-mouse IgG (H + L) secondary (Jackson Immuno Research Lab) at 1:200 dilution or Alexa Fluor™ 488 goat anti-mouse IgG (A11001, Lifetechnologies) at 1:400 dilution. For proliferation studies rat monoclonal antibody to Ki67 (SolA15; eBiosciences™, Invitrogen Cat#14–5698-80) was used at 1:100 concentration and primary was detected with goat anti-rat IgG (H + L) cy3 (AP136C; Millipore) secondary antibody at 1:200 concentrations. The sections were incubated overnight with primary antibody at 4°C in a humidified chamber. For BrdU staining, tissue sections were incubated in 2 N HCl at 37 °C for 30 min, neutralized in 100 mM sodium borate buffer pH 8.5 for 10 min at room temperature, washed with 1XPBS and blocked with goat serum blocking solution for 30 min. These sections were then incubated overnight at 4 °C with rat monoclonal antibody to BrdU [BU1/75(ICR1); abcam, Cat# ab6326] at 1:100 dilution. The primary antibody was detected with cy3 conjugated goat anti-rat IgG(H + L) cy3 (AP136C; Millipore) secondary antibody at 1:200 concentrations. The antibodies used in in-vitro targeting studies are as follows—monoclonal mouse anti-human pro-insulin supernatant (Developmental Studies Hybridoma Bank, Cat#GS-9A8) at 5µg/ml concentration, polyclonal rabbit anti-human glucagon (Dako, Cat#A0565) at 1:200 dilution, polyclonal rabbit anti-human amylase (Sigma, Cat#A8273) at 1:100 dilution.

### Targeted nanocarrier production and cargo encapsulation

- *End-group activation of Pluronic® F127* In order to conjugate antibody, terminal hydroxyl groups of pluronic chain were activated into amine reactive functional groups. For end-group activation, the hydroxyl end groups of the Pluronic®F127 chain were converted into amine-reactive p-nitrophenyl carbonate derivatives^22^. Briefly, Pluronic®F127 (MW 12,600, 2 g) was completely dissolved in anhydrous benzene (6 mL). A solution of p-nitrophenol chloroformate (p-NPC) in anhydrous benzene was gradually added to the stirred Pluronic® F127 solution, with p-NPC added in 3:1 molar excess of the terminal –OH groups present in Pluronic® F127. The mixture was stirred at room temperature for 24 h. The product was precipitated in 250 mL ice-cold diethyl ether; the residue was gravity filtered through Whatman filter paper and re-dissolved in benzene. The precipitation procedure was repeated 3 times to remove unreacted p-NPC altogether. After the third precipitation, the filtrate was thoroughly dried at room temperature in a vacuum desiccator to obtain the functional group activated Pluronic® F127-pNP (PF127- p-nitrophenyl ester). The degree of end group activation with p-nitrophenyl chloroformate was determined by UV analysis of the p-nitrophenol released from PF127-pNP after alkaline hydrolysis. The 1H NMR spectrum of the product was recorded at OHSU's NMR core facility, using a Bruker Advance 400 MHz high performance NMR spectrometer (Bruker Corporation, Billerica, MA) with deuterated water (D_2_O) used as a solvent to confirm successful end-group derivatization.
- *Encapsulation of Cargo* For proof-of-concept endocrine cell targeting studies, QDots were chosen as a model cargo and imaging agent, and these were encapsulated within the hydrophobic core of the nanocarriers. QDot-loaded end-group activated Pluronic® F127 nanocarriers [PF127(QD)-pNP] were prepared using a thin-film hydration method^23^. Briefly, 1.0 mg hydrophobic CdSe/ZnS (green fluorescent) or CuInS/ZnS (NIR) QDots (200 µL of 5 mg/mL QDot suspension in chloroform) were mixed in 20 mg PF127-pNP dissolved in 3 mL chloroform. The solution was stirred in dark for 4 h at room temperature, then sonicated for 5 min in a bath sonicator in the presence of ice. The solution was then transferred into a round bottom flask, and the organic phase was evaporated using a rotatory evaporator. The resulting thin film deposited at the wall of the round bottom flask was rehydrated with 3 mL HyPure Molecular biology grade (deionized, distilled) water (HyClone, Logan, UT). The round bottom flask was covered with aluminum foil and was kept on a shaker at 250 rpm for 2 h to obtain a nanocarrier solution, which was then centrifuged at 376 RCF for 15 min to remove any unencapsulated QDots. The supernatant was lyophilized to obtain a light-yellow colored (in case of green fluorescent QDot encapsulation) or light-brown colored (in case of near Infrared QDdot incapsulation) powder and stored at 4 °C. For harmine encapsulation, Pluronic F127/Harmine (100 mg/5 mg, w/w) were completely dissolved in 6 mL of chloroform by vigorous stirring at 40 °C over a magnetic hotplate stirrer in a glass scintillation vial, and later transferred into a round bottom flask and dried through rotary evaporation to form a thin film. The organic solvent was then removed by drying under a vacuum. The film was rehydrated in HyPure Molecular biology grade (deionized, distilled) water. Un-encapsulated harmine precipitate was removed by centrifugation, and the harmine loaded end-group activated nanocarrier [PF127(HM)-pNP] solution was lyophilized to obtain a white colored powder.
- *Antibody Conjugation* Lyophilized, cargo-loaded, end-group activated Pluronic® F127 [PF127(cargo)-pNP] was weighed (10 mg per animal) in a 1.5 ml Eppendorf tube. HPi1 or the negative control antibody, 6D2 in 1XPBS, were added dropwise to the PF127(cargo)-pNP to achieve a final ratio 1/100 ratio (wt/wt). The initial antibody concentrations were maintained at 2 mg/mL or more so that the volume of antibody solution added would not exceed 50 μL per 10 mg of polymer. The polymer was then incubated with the antibody at 4 °C for 30 min. Due to the sticky nature of the wet polymer, the solution of polymer and antibody was dissolved by vortexing and intermittent manual shaking, without using pipettes. The reaction volume was then increased to 100 μL by adding 0.1 M Sorenson's phosphate buffer at pH 8.0. The solution was then mixed using an end-over-end tube rotator overnight at room temperature^24^. The antibody conjugated PF127(cargo)-pNP was then centrifuged at 15,871 RCF for 15 min at room temperature to remove the unconjugated antibody. The supernatant was discarded, the pellet was washed three times with 1XPBS and then resuspended in 200 µL 1XPBS by vigorous pipetting and by ultrasonication to obtain a clear solution of PF127(cargo)-mAb nanocarriers. Control conjugates were prepared by conjugation to a negative control antibody that does not react with mouse tissue or normal human pancreatic cells. The final weight percent of antibody in the conjugates was determined using a BCA protein assay (Pierce Biotechnology, Rockford, IL), with BSA as a protein standard.

### Characterization of cargo-loaded nanocarriers

The size and morphology of the nanocarriers were determined by transmission electron microscopy (TEM; FEI T12 Tecnai™ T12 Spirit, Hillsboro, OR) at OHSU's Multiscale Microscopy Core. The nanocarrier dispersion was negatively stained with 1% uranyl acetate for TEM measurements. One 10 μL drop of an aqueous dispersion specimen was deposited on a carbon-coated TEM copper grid (PELCO® Grids, Ted Pella Inc., Redding, CA) with 300 mesh and allowed to dry in air for 2 min. The surface of the carbon film had previously been glow-discharged by exposure under plasma to render it hydrophilic.

Immediately before TEM sample preparation, the nanocarriers were vortexed for 60 s, vigorously pipetted, ultrasonicated for 10 min in the presence of ice, and vortexed again for an additional 10 s, which helped break down nanocarrier aggregates.

### Assessment of endocrine cell targeting in vitro

Specific targeting of pancreatic endocrine cells was investigated by treating enzymatically dispersed human pancreatic islets with antibody-targeted (PF127(QDot)-HPi1) and negative control antibody-conjugated nanocarriers (PF127(QDot)-6D2). One -thousand dispersed pancreatic islet equivalents were incubated with 2 mg of PF127(QDot)-HPi1 or negative control nanocarriers in 500 µL complete CMRL1066 growth media for 2 h at 37 °C. All the treatments were conducted in duplicate. Following incubation, the cells were centrifuged, and the cell pellet was washed with PBS three times to remove free nanocarriers. The cell pellet was dispersed in CMRL 1066 with 2% FBS, and the cells were stained with surface markers HPi2 (hybridoma clone HIC1-2B4) supernatant at 1:10 dilutiona mAb that reacts with all human pancreatic endocrine cells; HPa3 (hybridoma clone HIC3-2D12) supernatant at 1:10 dilution, a mAb that reacts with all non-beta endocrine cells; HPd3 (hybridoma clone DHIC5-4D9) supernatant at 1:10 dilution, a mAb that reacts with human pancreatic duct cells; and HPx1 (hybridoma clone HIC0-3B3) supernatant at 1:10 dilution antibody, a mAb that reacts with human pancreatic exocrine cells. After surface staining, the cells were fixed and permeabilized using the Intracellular Fixation & Permeabilization Buffer Set (eBiosciences, San Diego, CA; Cat#88-8824-00) and stained for various intracellular hormone markers to differentiate alpha, beta, duct, and acinar cells within the islet cell population. A confocal laser scanning microscope Zeiss LSM 780 (Carl Zeiss Microscopy GmbH, Germany) or Zeiss Axioskop 2 plus microscope was utilized for imaging. Based on marker expression, we counted the number of cells of each type that had associated QDots. A minimum of 100 surface marker positive cells was estimated from the treated cell samples that were stained for total endocrine, duct, or acinar surface markers. In this dataset, we further calculated the number of HPi2/pro-insulin or HPa3/glucagon double positives, which represented the percentage of nanocarrier internalizing beta and alpha cells respectively within the total counted endocrine cell population. The experiment was repeated using three different donor pancreatic islet preparations. Cells treated with the negative control nanocarriers were also stained and imaged to evaluate non-specific nanocarrier binding and/or uptake.

The total fluorescence signal associated with nanocarriers internalized by endocrine cells was also assessed. A minimum of 10 alpha or beta cells were counted for this measurement. These observations and calculations were made using Bitplane Imaris Scientific 3D/4D Image Analysis Software at OHSU's Advanced Light Microscopy Core. For this, 3D composite fluorescent images captured using the Z-stacking method on Zeiss LSM 780 confocal microscope were transferred to 3/4D image visualization and analysis software Imaris (Oxford Instruments, Abingdon, UK). Imaris software is integrated with distance-detection Matlab algorithms, which use fluorescence intensity data to detect objects in 3D space based on both intensity and size. First, surfaces were created using the "Surface Creation" module within the relevant fluorescent channel in the source image to segment the cells labeled for pro-insulin (beta cells) or glucagon (alpha cells). Utilizing fluorescence thresholding and masking tools found on Imaris, these surfaces were used to mask the green channel to segment the QDot loaded nanocarriers within the labeled cells only. More specifically, through an “intensity threshold” and a “number of voxels” filter, 3D objects were extracted from the original image file, using "Spot" analysis, which allows for quantification of the Qdot loaded nanocarriers internalized by the labeled cell. The objects defined as spots by the software are intracellular structures in which the QDot loaded nanocarriers localize. For quantification, statistics files were extracted and the pertinent information, i.e., the sum of average intensities within the green channel (nanocarriers) masked by red channel (intracellular hormone pro-insulin or glucagon) as a measure of endocrine cell subset type) was recorded. Here the sum of average intensities associated with the green channel masked by the red cy3 channel represents the sum of the average intensities related with the QDot loaded nanocarriers internalized by the labeled cells. This number correlates to the amount of QDot loaded nanocarriers internalized by the labeled cells. The extracted information was plotted using GraphPad Prism.

### Assessment of endocrine cell targeting in vivo

#### Islet xenograft model

For the human islet xenograft model, 1,000 human IEQ from cadaveric donors were transplanted under the renal capsule of anesthetized atleast 8 weeks old male NOD/LtSz-scid IL2R gamma null NSG Tg(RIP-HuDTR) mice. Human C-peptide levels were monitored on day 7, day 14, and day 21 post-transplantation using a Human C-peptide ELISA (ALPCO Diagnostics, Salem, NH) to ensure successful islet engraftment.

The animal care, handling, and all animal studies were performed following ARRIVE guidelines, in compliance with, and with approval of, the Oregon Health & Science University's Institutional Animal Care and Use Committee (IACUC, protocol# IP00001290).

#### Cell targeting study

Mice were intravenously injected via the retro-orbital sinus with 10 mg of nanocarrier [PF127(cargo)-mAb] in 100 µL 1xPBS. Whole-body fluorescence images were recorded at designated timepoints after intravenous administration of the nanocarrier formulation using a Xenogen IVIS® 200 Series instrument (Caliper Life Sciences, Hopkinton, MA) equipped with a 150 W Quartz halogen lamp and a 1 mW power scanning laser. The QDots were excited at 640 nm, with their emission detected using a 760 nm band pass filters. The fluorescence signal at the transplantation site was quantified using Living Image 4.0 software to calculate the flux radiating omnidirectionally from the region of interest (ROI) and graphed as radiant efficiency (photons/s/cm^2^/str)/(μW/cm^2^). To yield a standardized ROI for measuring the fluorescence signal, the same area of capture was used for each mouse. Fluorescence from a null or background capture area was measured and subtracted from each reading. The animals were humanely euthanized, and organs were harvested 48 h post-injection. Tissues were immediately embedded in OCT medium, frozen over liquid nitrogen, and stored at − 80 °C. Cryo-sections (5–10 μm) were mounted on glass slides and fixed in cold acetone for 10 min. Xenografts were located by immunofluorescence microscopy using fluorophore-labeled antibodies. Sections were mounted with Hoechst mounting media, and images were captured by confocal microscopy. N = 2 for each of targeted and negative control nanocarriers.

### In vivo harmine delivery

Human pancreatic islet xenografted mice were randomly selected to receive one of four treatments: antibody-targeted harmine-loaded nanocarriers PF127(HM)-HPi1, negative antibody control harmine-loaded nanocarriers PF127(HM)-HPDAC-6D2, harmine-HCl in 1xPBS or saline only. Harmine loaded nanocarriers were administered intravenously via the retro-orbital sinus once a day for 7 days (10 mg/kg/dose). Harmine-HCl was administered intraperitoneally every 12 h for 7 days (10 mg/kg/dose) as per Stewart et al.^13^. Animals were given BrdU in drinking water for 7 days. On the morning of day 8, animals were humanely euthanized, and kidneys were harvested, OCT blocks were prepared and stored at − 80 °C. Later, the sections were cut using a cryostat and immediately fixed in acetone. The sections were immunostained with HPi2 antibody to locate the xenograft. The sections were further immunostained for cell-proliferation markers BrdU or Ki67 to quantify proliferating cells.

### Cytotoxicity

The effect of nanocarriers on the viability of dispersed islet cells was evaluated using the CellTiter 96® Aqueous One Solution Cell Proliferation Assay (MTS, Promega, USA). This assay uses tetrazolium (3-(4,5-dimethylthiazol-2-yl)-5-(3-carboxymethoxyphenyl)-2-(4-sulfophenyl)-2H-tetrazolium, inner salt), a compound that is bio-reduced by metabolically active cells to a soluble formazan product. The quantity of formazan produced indicates the number of viable cells in the culture and is assessed colorimetrically by measuring the change in absorbance at 490 nm (Multiskan MMC microplate reader; Thermo Fisher Scientific). Enzyme dispersed islets were placed in a 96-well plate at a cell density of 10,000 cells per well. Then 100 μL of fresh culture media containing nanocarriers at concentrations of 50, 100, and 500 μg/100 μL was slowly added to the cells. After 2 and 4 h of nanocarrier exposure, the cell were collected in eppendorf tubes, centrifuged at 1000 rpm and the culture medium was removed. The cells were gently rinsed three times with PBS to wash off any free nanocarriers sticking to the cell surface. The cells were resuspended in 100µl fresh media, plated in 96 well format and 20µl MTS reagent was added to the cells. After 2 h of incubation with the MTS reagent, the cells were collected in eppendorf tubes, centrifuged at 1000 rpm and the supernatant was transferred to a new 96-well plate for read-out. The viability of the dispersed islet cells was determined by MTS reduction. Each treatment was carried out in triplicate, and cell survival was calculated with respect to untreated control cells.

## Results

### Synthesis and characterization of mAb-conjugated, cargo-loaded, Pluronic F127 nanocarriers

**Figure 2:**
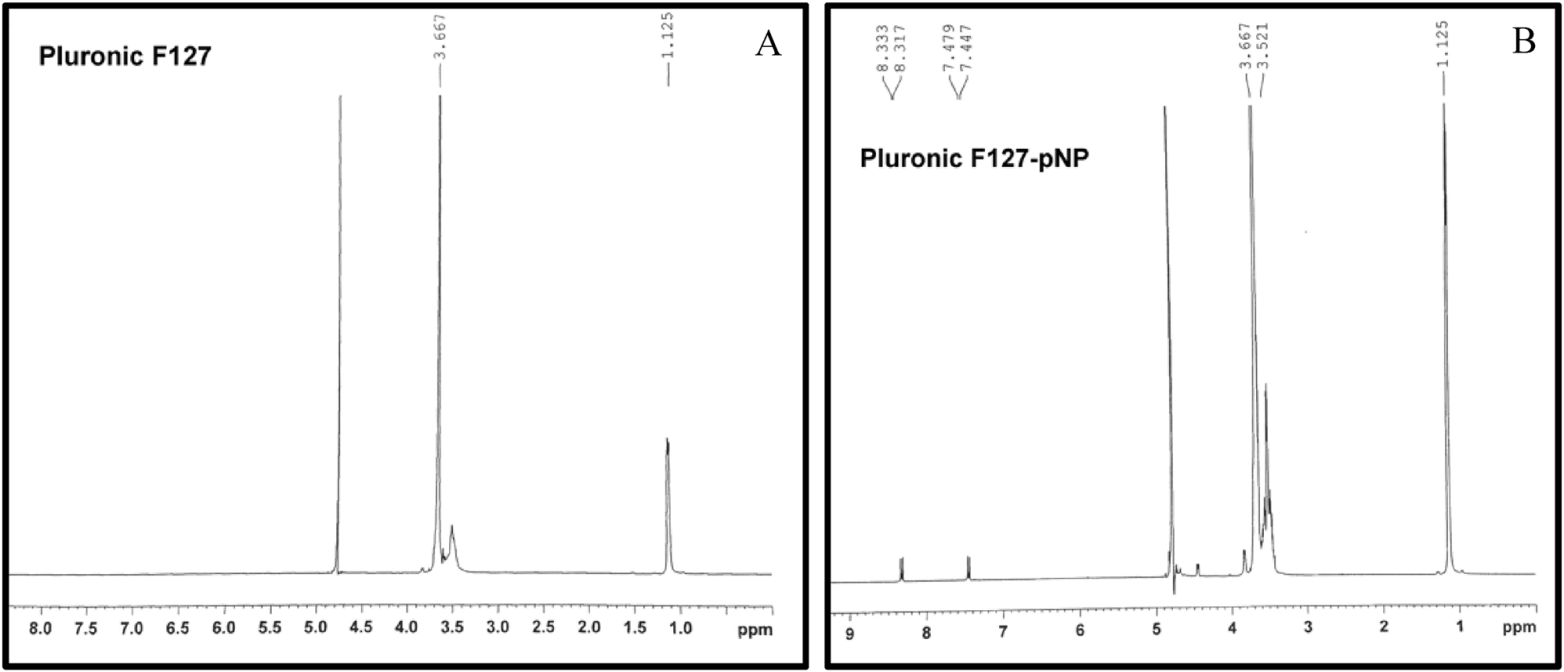
^1^H-NMR spectra of Pluronic-F127 (**A**) and p-nitrophenyl chloroformate activated Pluronic F127-pNP (**B**) in D_2_O. δ = 1.125 ppm (–CH_3_ protons in PPO blocks in Pluronic chain), δ = 3.5 ppm (–CH and –CH_2_ units of PPO block in Pluronic chain), δ = 3.66 ppm (–CH_2_CH_2_ units of PEO blocks in Pluronic chain), δ = 7.447/7.479 and 8.317/8.333 ppm (*p*-nitrophenyl doublet proton peaks).

Pluronic F127 is a tri-block copolymer comprised of a central hydrophobic polypropylene oxide (PPO) block flanked by two hydrophilic polyethylene oxide (PEO) blocks. The approximate lengths of the two PEO blocks are 100 repeat units for each PEO block, while the approximate length of the PPO block is 60 repeat units. The copolymer is activated with 1:3 molar excess of 4-nitrophenyl chloroformate to synthesize end-group activated PF127 (PF127-pNP). The activated product was analyzed with 1H nuclear magnetic resonance. Figure 2 shows the NMR spectra of Pluronic F127 and end group activated Pluronic F127-pNP in D_2_O. The peak intensities of various protons in the product are as follows 1H-NMR (D_2_O): = 1.1 (m, 3H, PPO, –CH_3_), 3.5 (m, 1H, PPO, CH), 3.5 (m, 2H, PPO, –CH_2_), 3.66 (m, 2H, PEO, –CH2), 7.4 (d, 2H nitrophenol, -CH) and 8.3 (d, 2H nitrophenol-CH) ppm. Peaks associated with aryl protons of the nitrophenyl groups (NO_2_–C_6_H_4_–, δ = 7.4–8.3 ppm) in the spectra of the product confirmed successful end group activation. The degree of activation was calculated by hydrolysis. For this quantification, activated pluronic is hydrolyzed in 0.1 M NaOH for 1 h, and absorbance of the hydrolysis product at 400 nm was calculated using the molar extinction coefficient of p-nitrophenol (18,100 M^-1^ cm^−1^) in 0.1 M NaOH. The degree of activation determined from the hydrolysis experiments was 57.3%. The peak intensity ratio of the aryl protons of the nitrophenyl groups (NO_2_–C_6_H_4_–, δ = 7.4–8.3 ppm) to the methyl protons of PF127 (–CH_3_, δ = 1.1 ppm) confirmed the degree of activation calculated from the hydrolysis data.

Pluronic based micelles have been well studied for their potential to efficiently retain hydrophobic small molecules in their hydrophobic PPO core^23^. The solubilities of the nanocarrier encapsulated hydrophobic drugs or cargo in an aqueous medium, therefore, is correlated to their encapsulation efficiency^23,25^. The encapsulation efficiency for Harmine and Qdots was measured using the following formula and was found to be ~ 60% and ~ 75%, respectively. BCA assay confirmed conjugation of ~ 67 µg antibody per 10 mg end group activated Pluronic F127-pNP, 67% of the total antibody used for the conjugation reaction.

$$Encapsulation\ Efficiency \left(\%\right)=\frac{Amount\ of\ cargo\ present\ in\ the\ nanocarrier}{Amount\ of\ cargo\ used\ for\ encapsulation}X100$$

**Figure 3:**
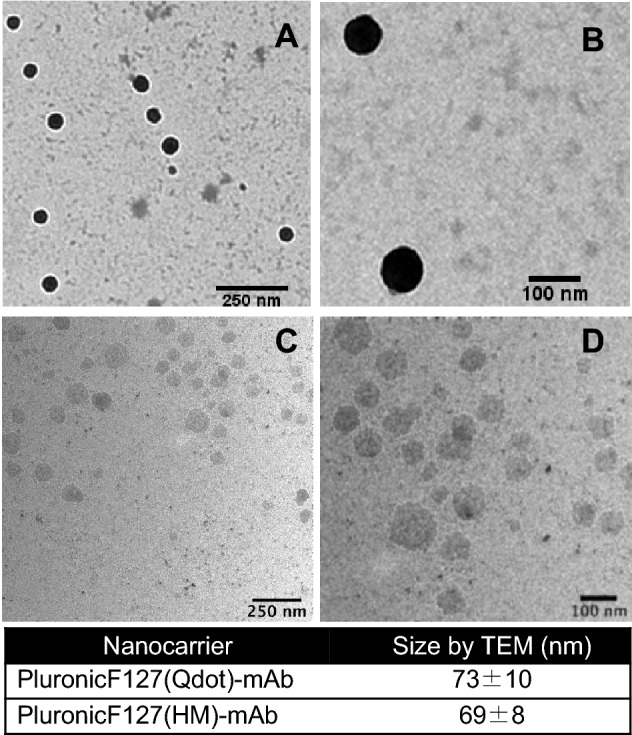
Antibody conjugated cargo loaded nanocarrier diameter was measured 73 ± 10 nm for QDot loading and 69 ± 8 nm for HM loading. (**A**,**B**) TEM images of Pluronic F127(Qdot)-mAb. (**C**,**D**) TEM images of Pluronic F127(HM)-mAb *HM* harmine, *TEM* transmission electron microscopy.

It has been previously observed that polymer based nanocarriers with diameters in the range of 10–100 nm successfully avoided uptake by the reticuloendothelial system (RES), which significantly increased the circulation half-life of the encapsulated drug^26^. Therefore, it was critical to estimate the average diameter of our cargo loaded antibody conjugated nanocarriers. The antibody conjugated nanocarriers PF127(cargo)-mAb were characterized for their sizes by transmission electron microscopy (TEM). Figure 3 shows transmission electron micrographs of the harmine or QDot loaded HPi1 antibody conjugated nanocarriers formulated in 1xPBS. TEM images confirmed that most antibody conjugated cargo encapsulating nanocarriers were found with an average size of 73 ± 10 nm for Qdot loading and 69 ± 8 for harmine loading. The qdot loaded nanocarriers appeared darker in contrast than harmine loaded nanopartciles because of the high electron scattering power of the quantum dots.

### In vitro targeting of endocrine cells by the nanocarriers

The ability of the HPi1 conjugated nanocarriers to bind specifically to the endocrine cells in human pancreatic islets was investigated in vitro using dispersed human pancreatic islet cells. The nanocarrier uptake by single cells was measured using fluorescent microscopy after incubating cells with nanocarriers for 2 h. Approximately 200,000 cells were incubated with the targeted (PF127(QDot)-HPi1) or negative control nanocarriers in CMRL growth media supplemented with 10% FBS. Following incubation, the cells were washed with 1XPBS to remove any nanocarriers not associated with cells. Further, 50,000 cells from each nanocarrier treated cell sample were immunostained for beta, alpha, acinar, and duct cell surface markers. Cells were also stained for the intracellular hormones pro-insulin, glucagon, and amylase.

**Figure 4:**
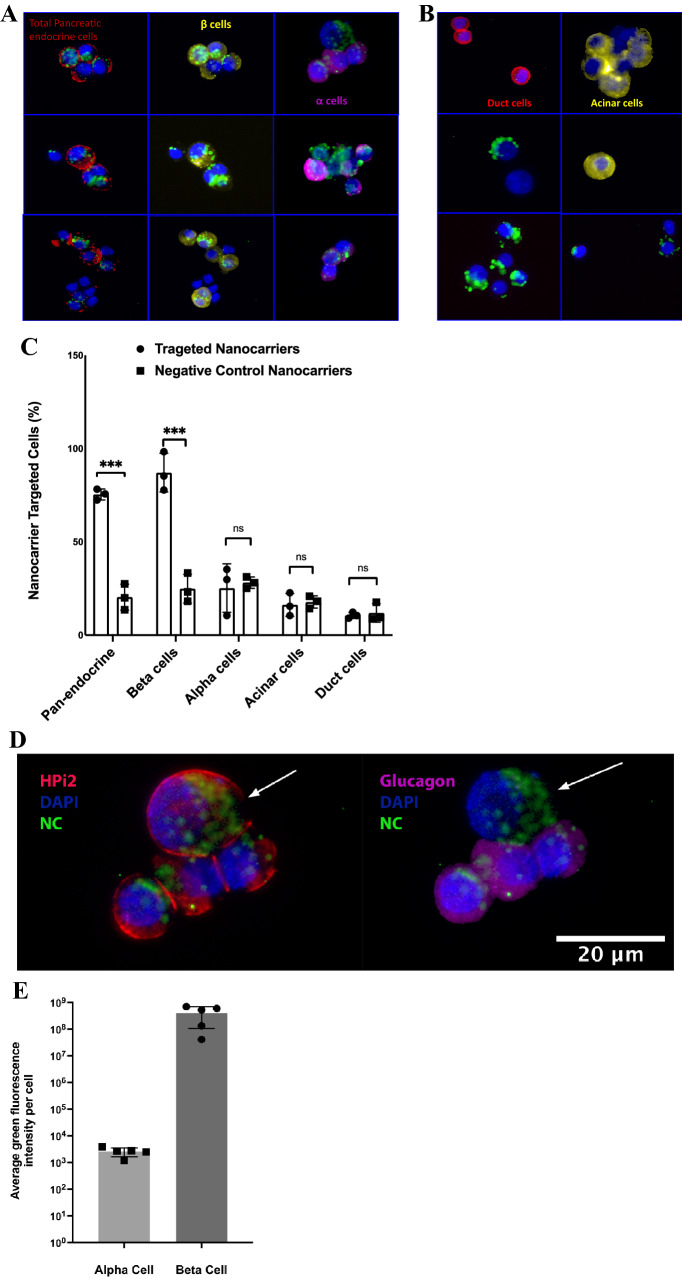
Representative images of dispersed human islet cells treated with qdot loaded targeted nanocarriers and co-immunostained with various surface and intracellular markers to determine the percentage of particular cell type internalizing targeted nanocarriers upon treatment in total endocrine, beta, alpha, acinar, and duct cells. Cells were counterstained with DAPI (blue). (**A**) Pancreatic endocrine cells showed uptake of targeted Qdot encapsulating nanocarriers. Endocrine cell markers; HPi2 (red), anti-pro-insulin (pseudo-colored yellow), anti-glucagon (pseudo-colored magenta) antibodies, targeted nanocarriers (green); (**B**) duct cells and acinar cells showed minimal uptake of the targeted nanocarriers. Duct cell marker; DHIC5 4D9 mAb (red) and anti-amylase antibody (pseudo-colored yellow ) For each cell-type evaluated, a minimum of 100 cells were counted. (**C**) Illustrates the percent of total endocrine cells, beta cells, alpha cells, acinar cells, and duct cells that internalized targeted nanocarriers. Results shown are the means ± standard deviation (n = 3). (**D**) HPi2 and anti-glucagon labeled cells; the cell indicated with arrow is most likely a beta cell which is HPi2 positive but anti-glucagon negative, (**E**) in accordance the data illustrates dramatically increased qdot signal associated with beta vs. alpha cells. *** indicates p-value < 0.001.

Figure 4A, B showed representative images from the in-vitro targeting experiments. As it can be seen in panel A, total endocrine cells stained by HPi2 (red) showed internalization of the QDot loaded nanocarriers, whereas non-endocrine (not stained; duct and acinar) cells did not internalize the nanocarriers. Similarly, pro-insulin positive cells (yellow) showed significantly higher uptake of the nanocarriers when compared to glucagon positive (magenta) cells which confirmed preferential uptake of the nanocarriers by beta cells. In contrast, Fig. 4B revealed that the non-endocrine cell population (duct and acinar cells) represented by cells labeled by duct cell marker DHIC5 4D9 (red) and acinar cell marker amylase (yellow) did not internalize the nanocarriers. The quantitative analysis revealed (Fig. 4C) that the targeted nanocarriers were selectively internalized by endocrine cells; 88% of the beta cells and 36% of the alpha cells showed nanocarrier uptake. By contrast, only 16% of the acinar cells and 9% of the duct cells showed nanocarrier uptake. Nanocarriers conjugated with negative control antibody did not exhibit selective internalization by endocrine cells and the percentages of nanocarrier internalizing total endocrine cells, as well as beta and alpha cells separately, were significantly lower than the HPi1 conjugated nanocarrier treated cells. The percentages of negative control nanocarrier internalizing duct and acinar cells were not significantly different from that obtained with the HPi1 conjugated nanocarrier treatment group. Thus, we conclude that the antibody conjugated nanocarriers selectively target endocrine cells, with a greater preference for beta cells. The total number of nanocarriers internalized by a particular cell type was measured by calculating the total fluorescence associated with the QDots encapsulated in the internalized nanocarriers. Figure 4D shows the representative image demonstrating that nanocarriers internalized by the alpha cells (magenta) were significantly lower than those internalized by the bets cells. In Fig. 4D, all the cells stained with HPi2 represent total endocrine cells; magenta stained cells represent alpha cell subset. Figure 4E shows the results of quantitative data analysis from 3 replicate experiments, which confirms that the number of nanocarriers internalized by the beta cells was significantly higher than that internalized by the alpha cells, differing by approximately 5 orders of magnitude (Fig. 4D,E).

### In vivo targeting of endocrine cells by the nanocarriers

To determine whether this targeting capability can also be achieved under physiological conditions, a single dose (10 mg Pluronic F127(QD)-pNP per animal) of targeted or negative control nanocarriers was administered intravenously via the retro-orbital sinus into NSG mice transplanted with human pancreatic islets under the kidney capsule.

**Figure 5:**
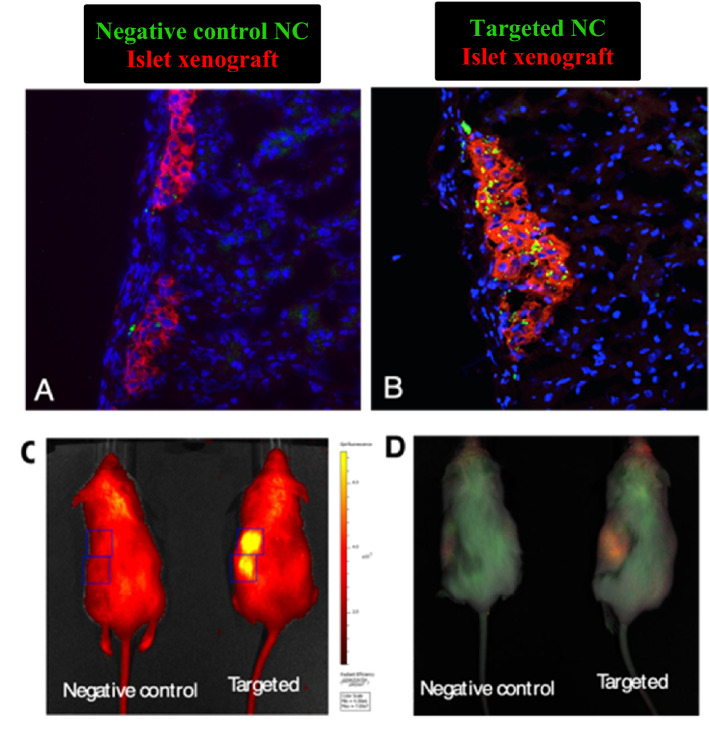
Representative images of human islet xenografts immunostained with HPi2 (red) antibody reactive to pancreatic endocrine cells to locate the graft in kidney sections of mice that received (**A**) QDot loaded negative control or (**B**) QDot loaded targeted, nanocarriers (green). The sections were counterstained with DAPI (blue). (**C**,**D**) Representative IVIS images from the Living Image 4.3.1 software of mice that received QDot loaded negative control (right) or targeted nanocarriers (left) under short isoflurane anesthesia. (**C**) Raw image before spectral unmixing (**D**) composite image obtained after multispectral imaging and spectral unmixing. The IVIS images were obtained at 48 h post-injection.

Figure 5C,D shows the data from whole body imaging of mice obtained from In Vivo Imaging System (IVIS). The results showed that the targeted nanocarriers effectively accumulated at the transplant site (kidney capsule), and the maximum signal associated with encapsulated NIR qdot was observed at 48 h post-injection timepoint. In contrast, negative control nanocarriers did not show any signal at the transplant site at this timepoint. The IVIS imaging helped identify the time of maximal nanocarrier uptake and xenograft harvesting to conduct ex vivo histological analysis. The ex vivo histological analysis performed on kidney sections revealed significant co-localization of targeted nanocarriers at human pancreatic islet xenografts. Figure 5A,B show the representative micrographs of the kidney tissue sections stained with HPi2 (red) to locate the xenograft. As shown in Fig. 5B, the images demonstrated significant nanocarrier accumulation at the xenograft location in the targeted nanocarrier group. By contrast, the QDot signal in the xenograft of mice injected with the negative control nanocarrier was negligible. Co-localization analysis was performed on the xenograft selected as a region of interest in the red fluorescence channel and QDot associated fluorescence in the green channel. The analysis revealed that the targeted nanocarriers were present in over 16% of the xenograft area compared to the negative control nanocarriers present in less than 2% of the xenograft area.

### In vivo targeted delivery of harmine

**Figure 6:**
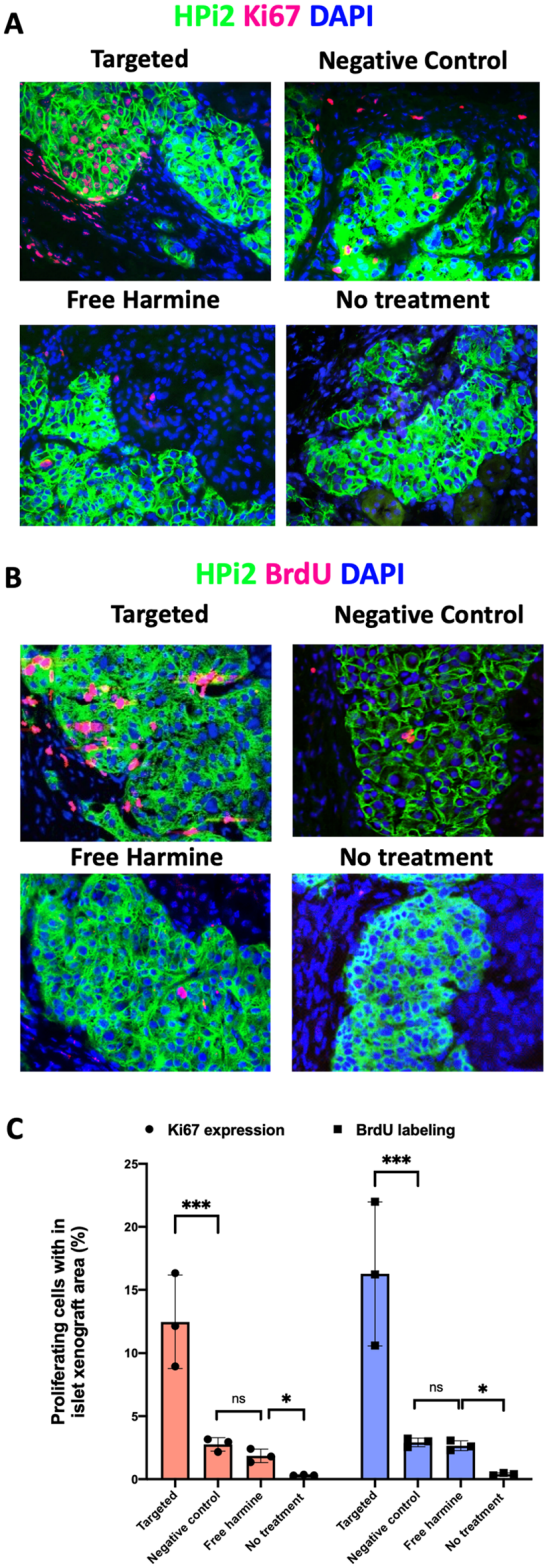
Representative human islet xenograft sections co-immunostained with HPi2 (green) and antibodies against (**A**) Ki67 (red) or (**B**) BrdU (red) a to measure proliferation in the transplanted islets upon treatment with harmine loaded targeted nanocarriers, harmine loaded negative control nanocarriers, harmine, and no treatment. The sections were counterstained with DAPI (blue). (**C**) Proliferating human islet cells within the xenograft from individual mice (n = 3) in each treatment group. Results are the means ± standard deviation for the percent proliferating cells of total HPi2 positive cells for each animal. Data were confirmed to be normally distributed by the Shapiro–Wilk normality test and analyzed using one-way analysis of variance (ANOVA) followed by Dunnett's test for multiple comparisons at α = 0.05. * and *** denotes p-value < 0.05 and p < 0.001 respectively.

Results from in vivo targeted harmine delivery studies (Fig. 6) revealed that along with efficient targeting of the pancreatic endocrine cells, the targeted nanocarriers successfully delivered the payload to the targeted cells, eliciting Harmine-induced cell proliferation. Figure 6A,B show the representative images of the tissue sections stained with the proliferation markers Ki67 and BrdU (red), respectively. The islet xenografts were identified using total endocrine cell reactive antibody HPi2 (green), and the sections were stained for nuclear stain DAPI (blue). Figure 6C summarized the quantitative analysis of the degree of proliferation in different treatment groups. The targeted nanocarriers loaded with harmine yielded Ki67 expression in approximately 12.47% of the pancreatic endocrine cells. By contrast, negative control nanocarriers did not exhibit selective targeting of endocrine cells and yielded 2.76% Ki67 positive cells. When harmine was injected in unencapsulated form (Fig. 6A, free harmine), 1.8% of the endocrine cells at the xenograft site showed proliferation measured by Ki67 expression. The degree of proliferation at the xenograft site was also confirmed using BrdU staining, and the results showed 16.27% BrdU labeled cells present at the xenograft in the targeted nanocarrier group. Harmine delivered encapsulated in the negative control nanocarriers (Fig. 6B, negative control) and unencapsulated form (Fig. 6B, free harmine) resulted in only 2.9% and 2.6% BrdU labeled cells in the xenograft, respectively. In the non-treatment control group, only 0.3% of the cell in the xenograft showed proliferation by Ki67 or BrdU staining.

### In-vitro evaluation of nanocarrier associated cytotoxicity

**Figure 7:**
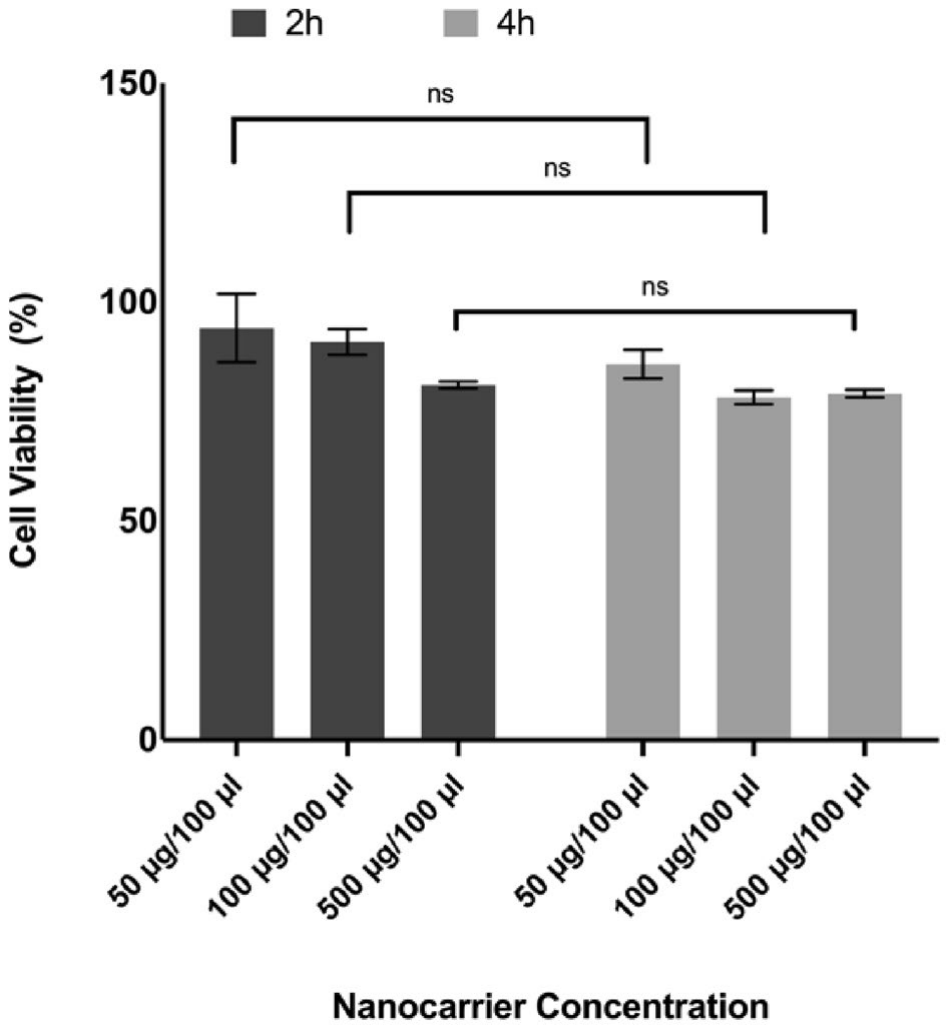
Effect of PF127(QD)-mAb nanocarrier formulation on the metabolic activity of dispersed human pancreatic islets, measured by the MTS assay. Cells were treated with nanocarriers at 50, 100, and 500 μg/100 µl concentration for 2 h and 4 h, in 10% FBS-containing cell culture media, and assayed 24 h post-treatment. Control cells were not treated with the nanocarriers; Data represent mean ± s.d. (n = 3).

To evaluate toxicity associated with the nanocarriers, we performed an MTS (3-(4,5-dimethylthiazol-2-yl)-5-(3-carboxymethoxyphenyl)-2-(4-sulfophenyl)-2H-tetrazolium cell proliferation assay on dispersed human pancreatic islets. The MTS assay is based on reducing a tetrazolium salt into a colored soluble formazan product by the mitochondrial activity of viable cells. As shown in Fig. 7, treatment with the nanocarriers did not significantly affect cell viability in vitro post 2 h or 4 h time points. For our in-vitro targeting experiments, we have incubated dispersed islet cells with the nanocarriers for up to 4 h. We were interested in learning about the nanocarriers' toxicity on the cells within this time frame. For this reason, the treatments were conducted for short periods, and the results represent the effect of nanocarriers on the islets cell viability only for short incubation times.

## Discussion

Type-1-Diabetes, also known as Juvenile Diabetes, is an autoimmune condition in which the body's immune system destroys the insulin-producing beta cells in the pancreas. In the absence of insulin, the body loses its capability of sensing elevated blood glucose levels and fails to maintain normoglycemia. The current standard of medical care for individuals with type 1 diabetes relies on insulin replacement therapy, which involves monitoring blood glucose levels multiple times a day and administering exogenous insulin via injections or continuous subcutaneous insulin infusion using insulin pumps^27^. Though insulin therapy in general effectively and safely maintains blood glucose levels close to normal in most individuals, its inability to closely mimic the endogenous pattern of insulin release leads to occasional hyperglycemia and related microvascular and cardiovascular complications^28^. Even if glycemic control is achieved, insulin administration enhances the risk of severe and potentially fatal hypoglycemia in type-1-diabetics^29^. An alternate strategy to counter insulin deficiency in type 1 diabetes is beta cell replacement therapy, which involves replacing dysfunctional or damaged beta cells with healthy insulin-secreting beta cells. Allogeneic pancreatic islet transplantation is one such strategy^30,31^. However, this treatment option requires long-term use of immunosuppressive transplant rejection drugs. Therefore, islet transplantation is the last resort for high-risk patients who experience severe hypoglycemic episodes. Moreover, due to limited donor islet availability and poor transplant durability, large-scale implementation of this procedure is not achievable at this time^30,32^. To overcome donor shortage, pancreatic islet xenotransplantation is currently being explored preclinically as an alternate beta cell replacement strategy^33^. Successful xenotransplantation has already been shown experimentally in various animal models^34^. However, the clinical application of islet xenotransplantation is far from being realized due to the similar hurdles that make allogenic transplantation inefficient, for example, immediate loss of islets due to instant blood mediated immune reaction, islet immunogenicity, and long-term need for immune suppression. In contrast, transplantation of beta cells derived from human embryonic stem cells (hESCs) or induced pluripotent stem cells (iPSCs) has been shown to overcome the challenges associated with graft rejection in allo- and xeno- transplantation as these beta cells are generated from patient's cells^35,36^. Clinical trials investigating the transplantation of hES cell-derived pancreatic progenitor cells to be matured to functional endocrine cells are currently underway in the US and Canada. Nevertheless, the study showed that the transplanted cells remain susceptible to the autoimmune attack^37^.

Another strategy to replenish the lost beta cell mass relies on the regeneration of endogenous beta cells from the existing beta cells in the pancreas of type-1-diabetics. Several signaling pathways and potential stimulants have been identified in this direction and shown to induce proliferation in the pancreatic beta cells^38^. Recently, Stewart et al. have reported a ground-breaking finding. The group has identified a small molecule mitogen, Harmine, a DYRK1A inhibitor, by high-throughput screening of small-molecule libraries. In vitro experiments demonstrated that harmine induced significant proliferation in human pancreatic beta cells by inhibiting DYRK1A signaling. Further, their work revealed that harmine increased islet cell mass and improved glycemic control using a diabetic marginal mass human islet transplantation model in mice^13^. It was further shown that when used in combination with TGF beta inhibitors, harmine induced the highest rate of proliferation ever observed in adult human beta cells^39^. This discovery identifies a path to a novel beta cell replacement strategy, where patients' beta cells could potentially regenerate new healthy insulin producing beta cells. One of the next key challenges in developing chemically induced endogenous beta cell regeneration therapies is to selectively deliver such proliferation-promoting chemical compounds directly to pancreatic beta cells. Cell selective targeted delivery would offer the potential of limiting exposure of unwanted cells and tissues to these chemicals and may thereby reduce unwanted proliferation and drug-associated toxicities.

The targeted nanocarriers discussed in this paper are self-assembled of Pluronic F127 amphiphilic block-copolymer, and the desired cargo was encapsulated within the hydrophobic core of the nanocarrier. To achieve endocrine cell specific targeting, the nanocarriers were futher conjugated with a targeting mAb HPi1 (hybridoma HIC0 4F9) that selectively reacts with cell surface molecules on all human pancreatic endocrine cells with a preference for the beta cells^14^. For proof of concept endocrine cell targeting studies, nanocarriers were loaded with a cargo of green fluorescent quantum dots (QDots) or a mixture of green and near infra-red (NIR) QDots. These cargos enabled the tracking of nanocarriers in vitro and in vivo, respectively. Results showed that approximately 75% of the total pancreatic endocrine cells and 88% of the beta cells internalized the HPi1 conjugated nanocarriers, whereas only 16% and 9% of acinar and duct cells showed nanocarrier uptake. The percentage of targeted nanocarrier internalizing exocrine cells is not significantly different from that observed in the case of negative control nanocarrier treated cells, implying that the uptake of targeted nanocarriers within the exocrine cells is mediated through non-specific internalization. In the targeted nanocarrier treated islet cell preparations, though approximately 36% of the total alpha cells showed internalization of the nanocarriers, the amount of nanocarriers internalized in the beta cells was 5 order of magnitude higher than that internalized in the alpha cells. This result is not unexpected because the antibody HPi1 used as a model targeting moiety in our experiments has earlier been shown to label all endocrine cells, including but not limited to insulin-expressing β cells. However, the reactivity of HPi1 with beta cells was found to be higher than alpha cells by flow cytometry (Dorrell & Streeter et al, unpublished). This attribute of the antibody was reflected in our experiments, too, and addresses the internalization of the HPi1 conjugated targeted nanocarriers in alpha cells. The higher reactivity of HPi1 and the higher level of internalization of the HPi1 conjugated nanocarriers with the beta cells as compared to alpha cells indicate that antibody reactivity and the uptake of targeted nanocarriers within specific cells type might depend on the cell surface density of the targeted antigen and overall cell morphology. However, this is a matter of further investigation.

Similarly, in-vivo experiments on human islets transplanted in mice showed significantly higher accumulation of the targeted nanocarriers measured by whole body imaging and histology than that observed in the case of negative control nanocarriers.

Further, we used harmine as a model cargo in the targeted drug delivery studies. As discussed above, harmine has already been shown to induce quantifiable biological responses in beta cells. In addition, being highly hydrophobic, harmine favors more compact packing within the hydrophobic micellar core of pluronic, enabling a high degree of encapsulation. In our studies, Pluronic F127 micelles encapsulated approximately 60% of the harmine. The results showed that harmine loaded targeted nanocarriers induced proliferation in up to 12% (by Ki67 staining) and 16% (by BrdU labeling) cells in the islet xenografts.

Our findings were consistent with the previous investigations published by Moore et al. demonstrating targeting islet cells in the mouse pancreas by an intact mAb conjugated with radionuclide for nuclear imaging^20^. The antibody used in this study was β-cell–specific monoclonal antibody IC2. This report is particularly instructive in that the antibody used was an IgM (approximate molecular mass of 900 kD). The Cryo-AFM and Cryo-TEM diameters of IgM, in the range of 35–50 nm^40,41^, suggest that the vasculature within islets supports the passage of large biomolecules. The presence of functionally specialized fenestrated islet endothelium is thought to enable the transport of macromolecules out of the vasculature. Therefore, the antibody conjugated cargo (QDots or harmine) encapsulating Pluronic F127 nanocarriers used in this work, having an average size of 73 ± 10 or 69 ± 8 nm, were favorable to achieve the in vivo targeting of pancreatic islets.

Further, in our in-vivo endocrine cell targeting studies, IVIS imaging at various time points after intravenous injection revealed that the Qdot loaded targeted nanocarriers showed maximum accumulation, 48 h post-injection time point at the transplant site, observed as a bright signal associated with encapsulated NIR qdot. At this time point, the negative control nanocarriers did not show any signal in IVIS images. Negative control nanocarriers presented faint signal in IVIS images only after 72 h (data not shown). This implies that the favorable size of this pluronic based nanocarrier assembly prolonged the circulation time of qdot in the blood. Moreover, the presence of fenestrated endothelial cells allows for the passive accumulation of the nanocarriers within the islet xenograft regardless of the targeting antibody. However, targeting antibody facilitated active binding of the nanocarriers to xenografted pancreatic endocrine cells. In contrast, negative control nanocarriers circulated without actively binding to the xenograft and showed minimal non-specific retention over time due to passive accumulation. Ex-vivo histology further confirmed the observations from IVIS imaging. The kidney tissue sections revealed that at 48 h post-injection timepoint, the targeted nanocarriers significantly co-localized at the islets transplanted under the kidney capsule. In contrast, minimal co-localization was observed in the negative control nanocarriers treatment group at the 48 h time point.

We further investigated whether the selective delivery of harmine is capable of inducing proliferation in the targeted cells in the same mouse model. This investigation determined not only the cargo delivery capability of the nanocarriers but also the delivery of the functionally active cargo capable of inducing a quantifiable biological response only at the target site without compromising its mitogenicity. For drug delivery studies, harmine was loaded in the nanocarriers by the thin-film evaporation method. Harmine delivery by targeted nanocarriers yielded approximately 12% Ki67 positive cells and 16% BrdU labeled cells within the islet xenografts. In contrast, when harmine was delivered in unencapsulated form, 1.85% and 2.65% of the total endocrine cells were found to be positive for Ki67 expression and BrdU labeling, respectively. This level of proliferation induced by unencapsulated harmine at a similar dosage is consistent with the studies reported earlier by Stewart et al. Proliferation induced by the negative control nanocarriers within the islet xenografts was found to be not statistically significantly different from that observed when harmine was given unencapsulated. This observation suggests that the proliferation induced by harmine when delivered via non-targeted nanocarriers is due to the minimal non-specific uptake of the non-targeted nanocarriers at the transplant site. We have observed that the total number of proliferating cells measured by the BrdU staining method was consistently higher than the measurement made by the Ki67 staining method. This difference may be explained by the fact that in our experiments, the mice accessed BrdU in drinking water during the whole course of the treatment (1 week), allowing BrdU uptake by the total number of cells that proliferated during the week. By contrast, Ki67 expression revealed the cells that were proliferating at the end-point.

Compared to the unencapsulated Harmine or Harmine given in negative control nanocarriers, Harmine given in targeted nanocarriers induced a six–seven-fold greater increase in proliferation of endocrine cells, measured by both Ki67 expression and BrdU labeling of xenografted islet cells. At the base level, 0.3–0.4% of the total endocrine cells in the xenograft were found to be proliferating in the untreated control mice. This data concludes that harmine given in the encapsulated and antibody targeted nanocarrier formulation is significantly more efficacious than when given in unencapsulated or encapsulated but untargeted formulations.

Harmine dosage of 10 mg/kg in our experiments was decided based on the previously reported study, which demonstrated quantifiable proliferation levels in the islet xenografts without causing any toxic side effects in the animals^13^. In our experiments, though we have not observed any drug-associated toxicity in animals at the given harmine dosage, we have observed prominent unwanted proliferation in the cells present outside but closely associated with the islet xenograft in the targeted nanocarrier treatment group. Bani et al. have shown earlier that human pancreatic islets abundantly express p-glycoprotein and indicated the possibility that these p-glycoproteins play an essential role in regulating the secretory function of the human pancreatic islets and thereby maintaining the composition of the extracellular fluids and their intracellular microenvironment^42^. We anticipate that the undesirable cell proliferation in the close vicinity of the xenograft could be due to the p-glycoprotein mediated efflux of harmine in the nearby cells.

Harmine’s highly hydrophobic nature facilitated higher encapsulation efficiency within the nanocarriers. Along with this, the longer circulation time of the nanocarriers and active binding to the endocrine cells mediated by the targeting antibody describe the high biological efficacy of harmine at the target site. On the other hand, encapsulation and targeting present the opportunity to reduce the daily dosage or frequency of administration of the drug loaded nanocarriers. We anticipate reducing the daily dosage or frequency of the drug loaded nano carriers might help mitigate the unwanted proliferation.

In practical applications, the effective therapeutic response from a drug depends on the optimum plasma concentration of the drug obtained and maintained over time. Without achieving this ideal concentration, the drug won't be effective^43^. However, most drugs are prone to enzymatic degradation while in the circulation^42^, or they get cleared from systemic circulation too soon before being effective at the targeted site. If the drug's circulation time is very short, it is given at a higher dosage and multiple times to achieve and maintain the ideal plasma concentration. This is particularly true for a lot of anti-cancer drugs. A higher concentration of anti-cancer drugs in the system is known to cause adverse effects, toxicities, and immune responses. Similarly, in the case of harmine and other mitogenic small molecules- it is crucial to deliver these molecules to the desired site to avoid unwanted proliferation in other cells and tissues. Therefore the probability of significant dose reduction to achieve desired therapeutic efficacy when the drug is given in targeted nanocarrier formulations is highly valuable. Once such drugs are encapsulated within a nanocarrier, and the nanocarriers are guided to the target site via antibodies conjugated on their surface, they steer clear of other tissues and organs. Upon encapsulation, these drugs will escape enzymatic degradation, which will increase the drug's circulation half-time. Altogether, drug delivery via targeted nanocarriers potentially reduces drug dosage to elicit equally effective therapeutic response while mitigating drug associated toxic response.

Based on our findings, we anticipate that the platform technology described here will have application in drug discovery, in the in vivo evaluation or validation of candidate drugs; in the evaluation of different endocrine cell targeting moieties (e.g., antibodies, antibody fragments, aptamers, etc.); and therapeutic drug delivery and/or diagnostic imaging in individuals with diabetes or prediabetes.

## Supplementary information

Supplementary Information.

## Data Availability

The datasets used and/or analyzed during the current study available from the corresponding author on reasonable request.
