# Supplementary material for: Targeted delivery of harmine to xenografted human pancreatic islets promotes robust cell proliferation
Source: Sci Rep. 2022 Nov 9;12:19127. doi: 10.1038/s41598-022-19453-5 (PMC9646720; doi:10.1038/s41598-022-19453-5)
Supplement: Supplementary file 1 — Supplementary Information. [file 41598_2022_19453_MOESM1_ESM.pdf]

Supplemental Information

Targeted delivery of harmine to xenografted human pancreatic islets promotes robust cell proliferation

Swati Mishra, Philip R. Streeter

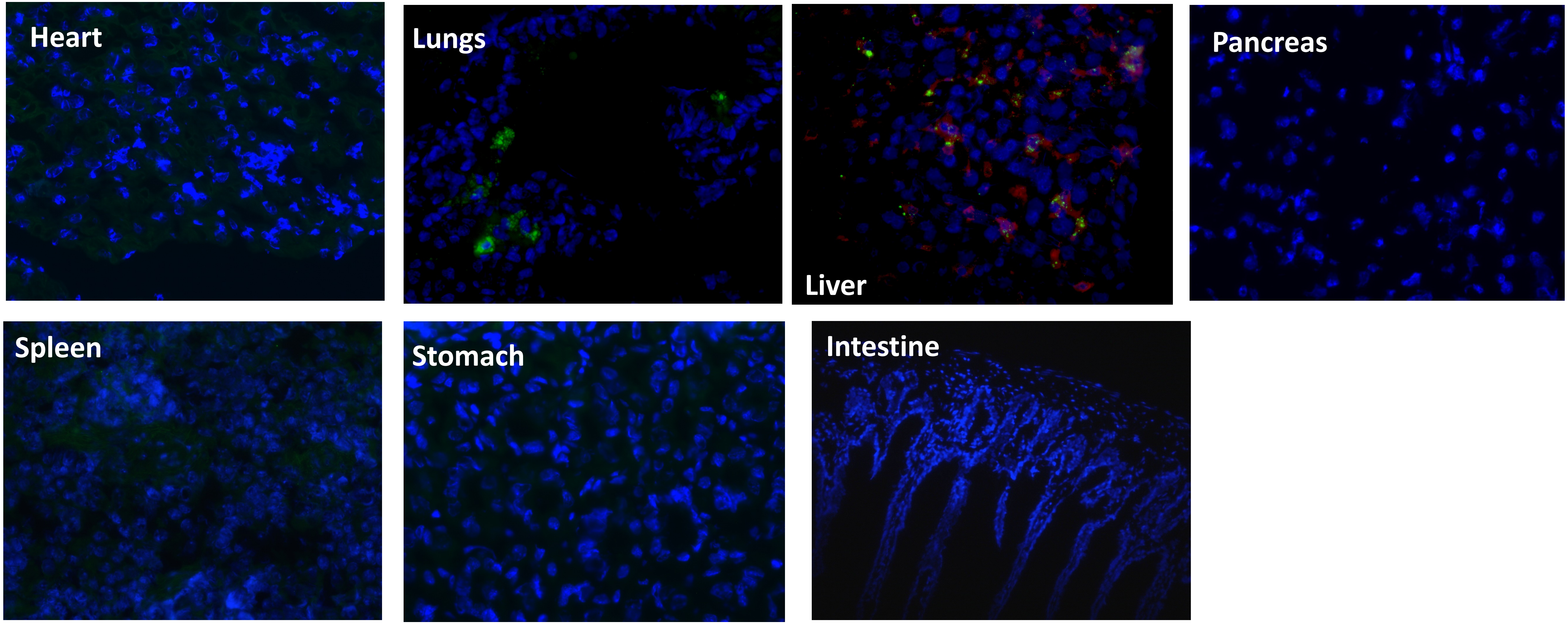

Tissue sections from other organs
Targeted (harmine/Qdot) loaded nanocarrier treatment group

Red-CD45, Green-Nanocarrier(Qdot) (40X)

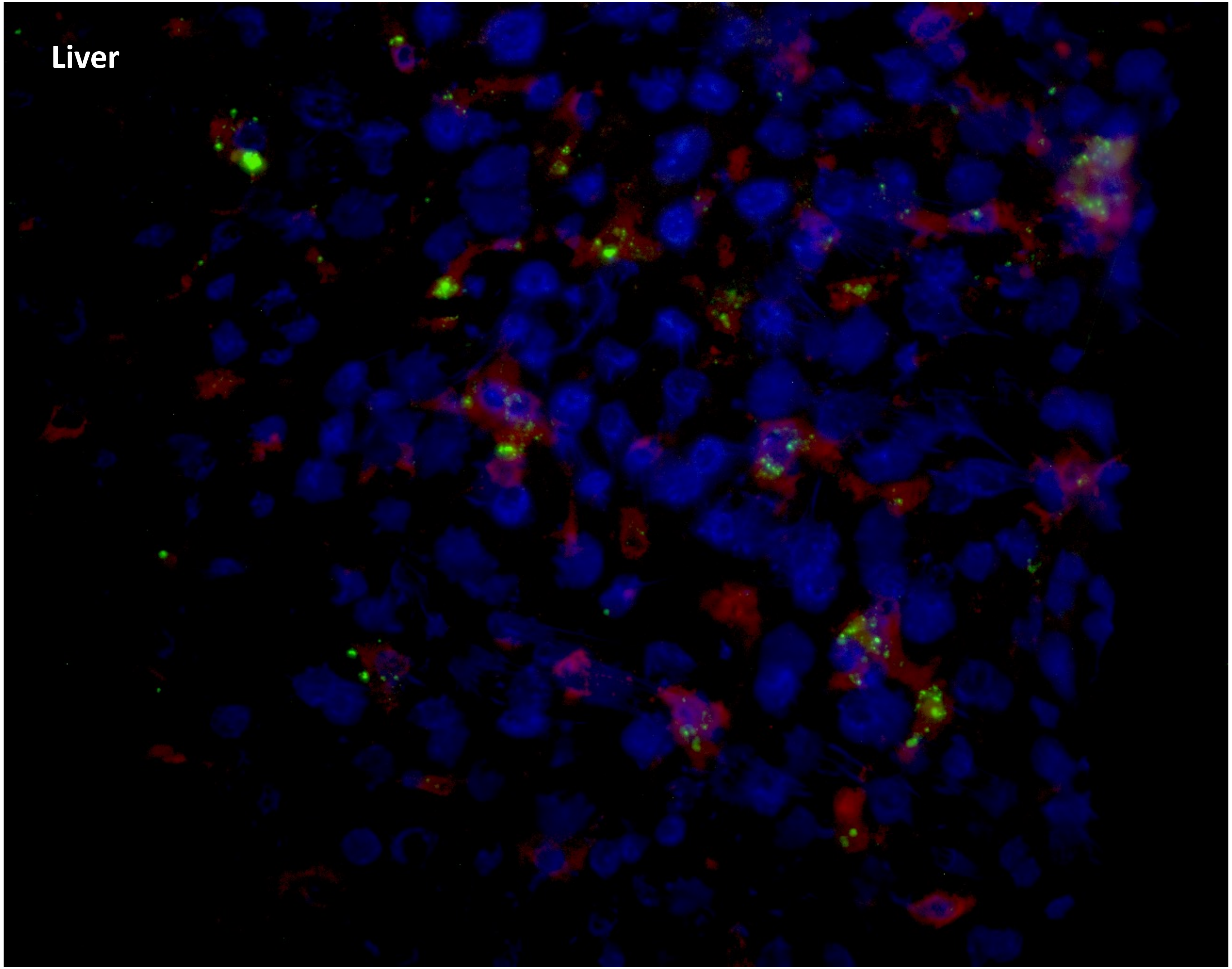

Red-F4/80, Green-Nanocarrier (Qdot) (40X)

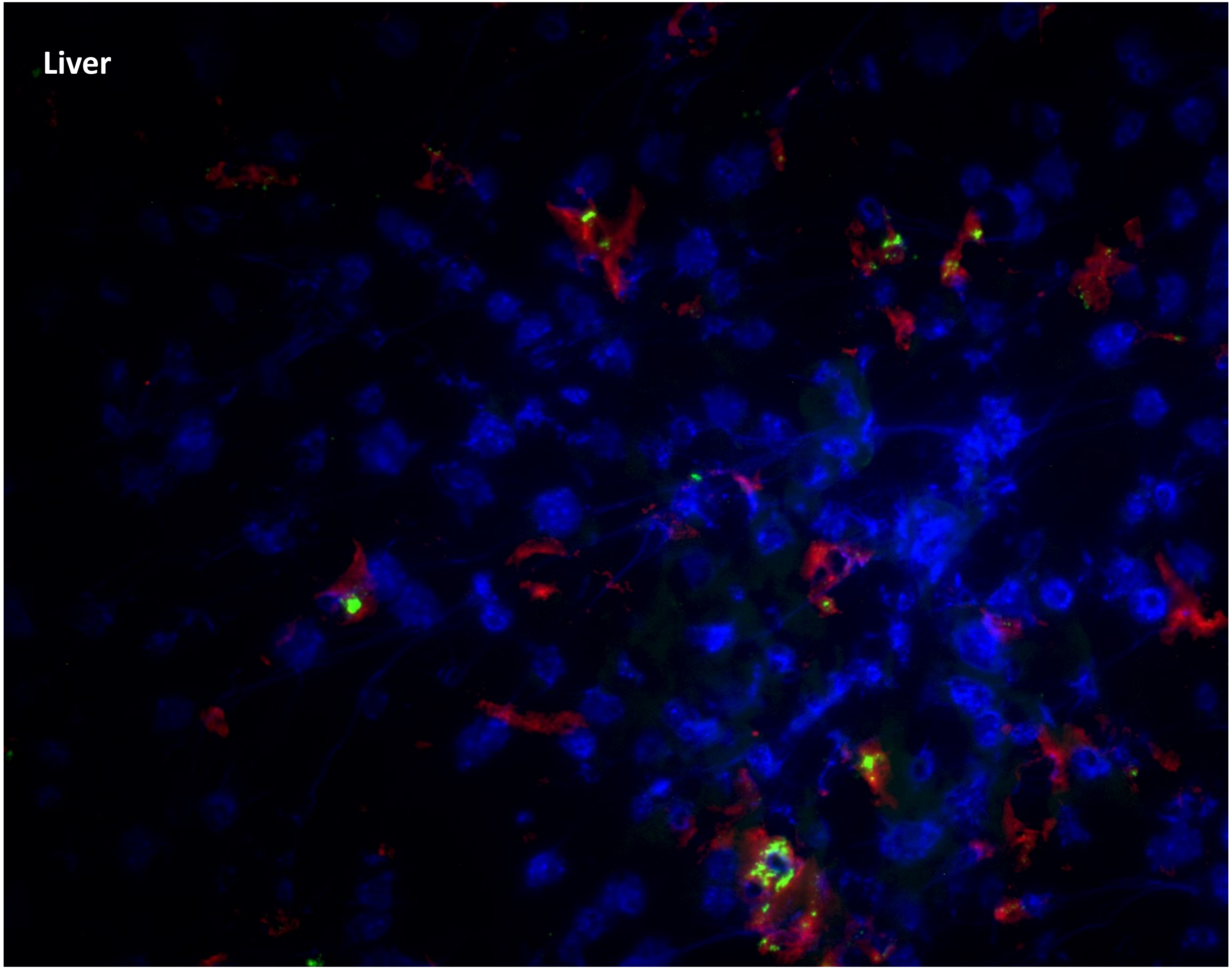

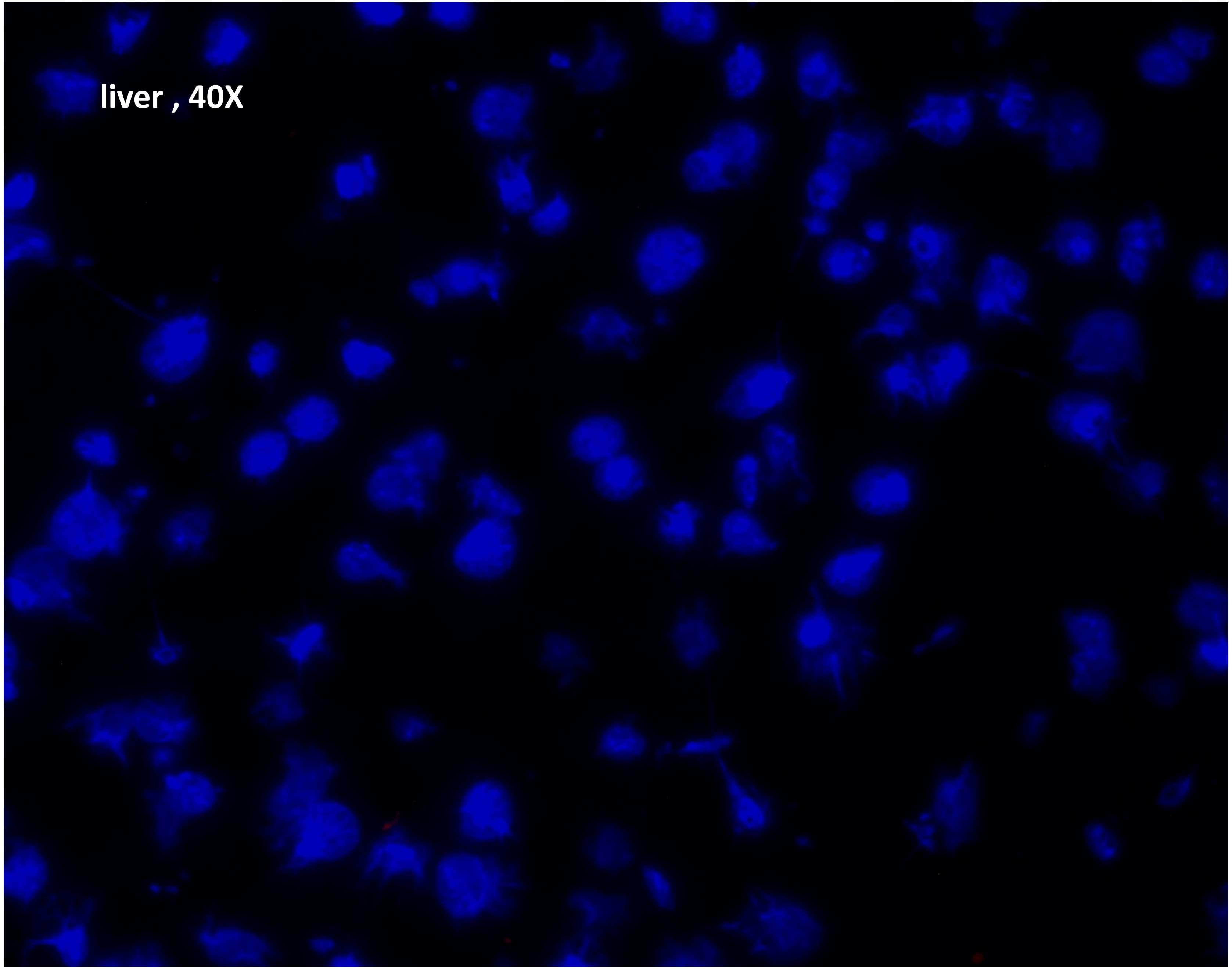

Stained for Blue DAPI and Ki67 (to observe proliferation in liver)

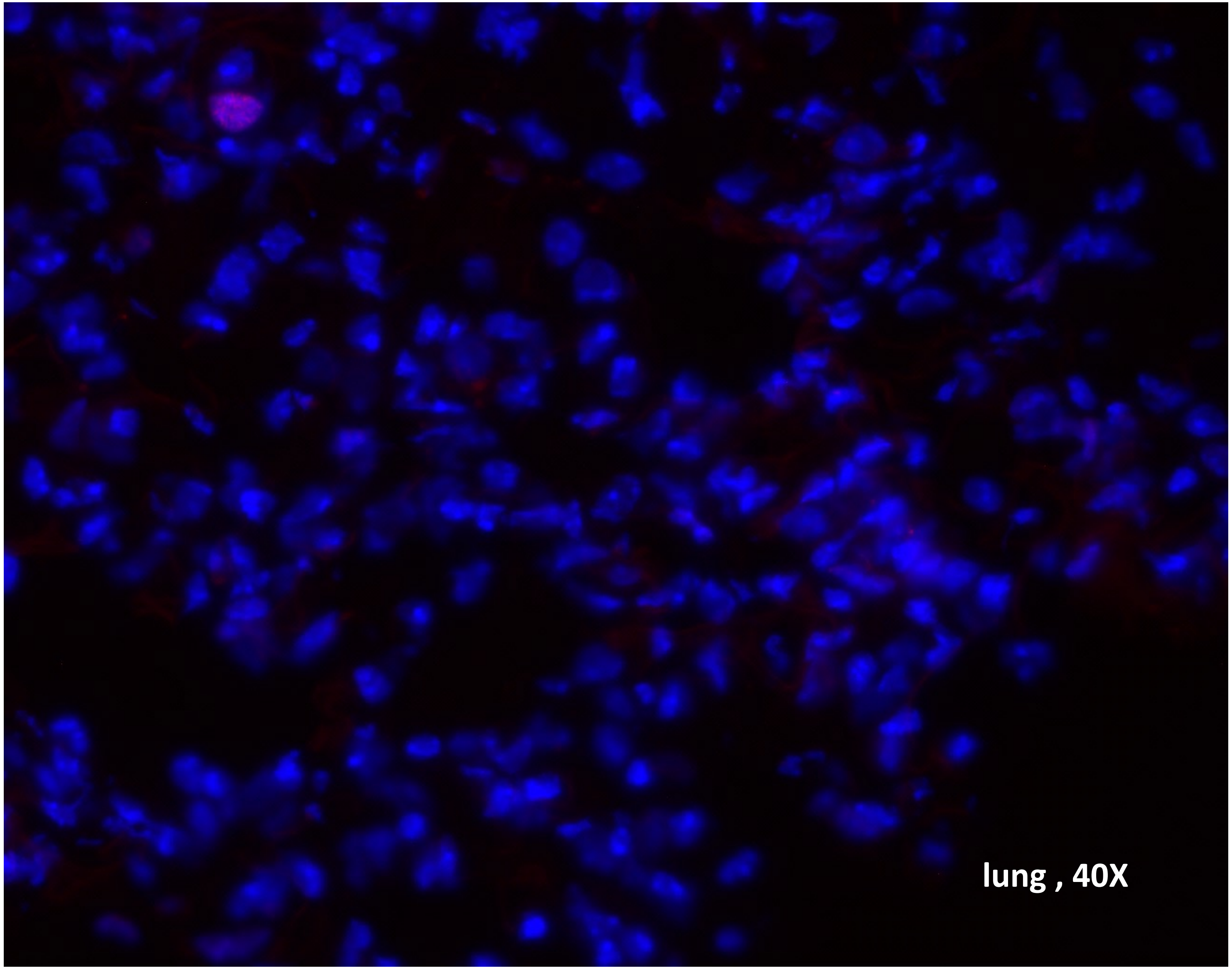

Stained for Blue DAPI, Magenta Ki67 (to observe proliferation in lung)
